# Comparative Analysis of Chloroplast Genomes of “Tiantai Wu-Yao” (*Lindera aggregata*) and Taxa of the Same Genus and Different Genera

**DOI:** 10.3390/genes15030263

**Published:** 2024-02-20

**Authors:** Yujie Shi, Zhen Chen, Jingyong Jiang, Xiaobai Li, Wei Zeng

**Affiliations:** 1 Zhejiang Provincial Key Laboratory of Plant Evolutionary Ecology and Conservation, College of Life Sciences, Taizhou University, Taizhou 318000, China; shiyujie@tzc.edu.cn (Y.S.); chenzh@tzc.edu.cn (Z.C.); 2 Institute of Horticulture, Taizhou Academy of Agricultural Sciences, Linhai 317000, China; jjy5971@163.com; 3 Institute of Horticulture, Zhejiang Academy of Agricultural Sciences, Hangzhou 310021, China; lixiaobai@mail.zaas.ac.cn

**Keywords:** Lauraceae, *Lindera*, chloroplast genome, comparative analysis, phylogeny

## Abstract

*Lindera aggregata* is a species of the Lauraceae family, which has important medicinal, economic and ornamental values. In this study, we sequenced, assembled and annotated the chloroplast genome of *L. aggregata* and reannotated and corrected eight unverified annotations in the same genus. The chloroplast genomes taxa from Lindera and from different genera of Lauraceae were compared and analyzed, and their phylogenetic relationship and divergence time were speculated. All the 36 chloroplast genomes had typical quadripartite structures that ranged from 150,749 to 154,736 bp in total length. These genomes encoded 111–112 unique genes, including 78–79 protein-coding genes, 29–30 tRNA and 4 rRNA. Furthermore, there were 78–97 SSRs loci in these genomes, in which mononucleotide repeats were the most abundant; there were 24–49 interspersed repeats, and forward repeat types were the most frequent. The codon bias patterns of all species tended to use codons ending with A or U. Five and six highly variable regions were identified within genus and between genera, respectively, and three common regions (*ycf1*, *ndhF-rpl32* and *rpl32-trnL*) were identified, which can be used as important DNA markers for phylogeny and species identification. According to the evaluation of the Ka/Ks ratio, most of the genes were under purifying selection, and only 10 genes were under positive selection. Finally, through the construction of the evolutionary tree of 39 chloroplast genomes, the phylogenetic relationship of Lauraceae was clarified and the evolutionary relationship of *Lindera* was revealed. The species of genus *Lindera* experienced rapid adaptive radiation from Miocene to Pleistocene. The results provided valuable insights for the study of chloroplast genomes in the Lauraceae family, especially in the genus *Lindera*.

## 1. Introduction

Lauraceae is one of the most important woody families in the flora, with about 50 genera and 3500 species [1,2]. The genus *Lindera* is one of the most core genera, with approximately 100 species distributed in tropical, subtropical and temperate regions of Asia and the Midwest of the USA [3]. In China, there are more than 40 species, accounting for 46% of the total genus [4]. Most of the plants of the genus *Lindera* have a special smell and can be used for cooking and medical purposes [4,5]. Among them, *L. aggregata* is a kind of plant with great medicinal value in this genus, which was widely used in the traditional medicine industry as early as 2000 years ago. The dry tuberous root of *L. aggregata* is the main part of the medicine [6], and it is also called “Wu-Yao” in Chinese because the root is black–brown. *L. aggregata* is mainly produced in eastern, central, southern and southwestern blocks of China, such as in Zhejiang, Jiangxi, the Hunan provinces and other places. Among them, the *L. aggregata* produced in Zhejiang Tiantai is of the best quality, which is also known as “Tiantai Wu-Yao”. It is the authentic origin of *L. aggregata* since the Song Dynasty [4]. Furthermore, *L. aggregata* contains a large number of isoquinoline alkaloids [7], flavonoids [8], sesquiterpene lactones [9] and polyphenols [10], with anti-cancer, anti-inflammatory, antibacterial, antioxidant and other activities [6,11,12,13], that can be used to treat chest pain, abdominal pain, inflammation, frequent urination, dysmenorrhea, rheumatic diseases and other diseases [6,14]. According to historical records, during the Tang Dynasty in China, Master Jianzhen, a monk, cured the disease of the Empress Dowager Guangming of Japan with *L. aggregata* and it was regarded as the “Elixir of Life”. Although its medicinal value has been fully studied and recognized, there are few studies on its genetic diversity and phylogeny.

The chloroplast is the place in which photosynthesis occurs, and it has a relatively independent genetic system [15]. The chloroplast genomes of angiosperms are usually 120–180 kb in size and have highly conserved circular quadripartite structures. Two inverted repeats (IRs) separate large single copy (LSC) and small single copy (SSC) regions [16]. The differences among chloroplast genomes of different species are mainly reflected in the length and direction of IR regions. Chloroplast genes encode 100–130 genes, which are usually divided into four categories: (1) self-replication genes, which are related to transcription and translation; (2) photosynthesis genes, which encode photosynthesis-related proteins; (3) other genes, related to the synthesis of amino acids, fatty acids and other substances and (4) unknown functional genes. Among them, photosynthesis-related genes are distributed in the LSC region and SSC region. Although the chloroplast genome structure is relatively conservative, gene transfer, recombination and transposition events may occur, and there may be hot spots of mutation [17]. At present, the chloroplast genomes of several species of the genus *Lindera* have been reported [18,19,20]. The length of these complete genomes ranges from 152,478 to 153,679 bp. It is reported that some gene regions show a high level of variation in the DNA markers and intra-genus studies of the Lauraceae family, such as eight gene spacers (*accd-psaI*, *ndhC-trnV*, *ndhF-rp32*, *psbm-trnD*, *rp32-trnL*, *rp14-rps8*, *rps3-rps19* and *rps16-trnQ*), three gene fragments (*rbc1*, *ycf1* and *ycf2*) and three introns (*clpp*, *ndhA* and *trnG-UCC*) [21,22,23,24,25,26]. These complete chloroplast genome sequences are of great value in understanding the phylogenetic relationship and species evolution among similar groups of plants [16]. The emergence of a new generation of high-throughput sequencing technology greatly reduces the sequencing prices and improves the sequencing throughput, which makes it possible for large-scale chloroplast genome sequencing. The study of phylogenetic genomics based on high-throughput sequencing has become an important tool for a comprehensive understanding of biological evolution [18] and has been widely used in molecular breeding, phylogeny, endangered species protection and other fields.

Up to now, the research on *L. aggregata* is mainly focused on its chemical composition and pharmacological action [4,5], but there are few studies on its genetic diversity, evolution and phylogeny. Although the chloroplast genome of *L. aggregata* has been reported, the samples collected are mainly from Zhejiang Hangzhou or Yunnan [20,27], and the trueborn origin region (Zhejiang Tiantai) has not been reported, and the previous research content is also only a simple characteristic description. It is not included in Lauraceae or *Lindera* as a whole for comparison, and the chloroplast genome information is not fully exploited. Furthermore, *Laurus*, *Lindera* and *Litsea* are closely related, and they all have similar morphological characteristics, such as dioecious, umbels wrapped in a large involucre, anthers all introverted and two stalked glands in the third round [28,29]. Especially in the genus *Lindera*, the morphological characteristics of different species are very similar, which may lead to difficulties in identification and misconstruction of phylogenetic relationships. Therefore, the phylogenetic relationship and genomic information of *L. aggregata* in the genus *Lindera* and among different genera of Lauraceae still need to be further improved.

In this study, we sequenced, assembled and annotated the chloroplast genome of *L. aggregata* and re-annotated to modify a large number of these taxa’s unverified chloroplast genomes in the Lauraceae family. The characteristics of their chloroplast genomes were compared and analyzed from within genus and between genera; their chloroplast genome variations were quantified and candidate molecular markers were obtained. The phylogenetic relationship of Lauraceae was discussed and the divergence time was estimated, which laid a foundation for further study of genetic diversity and the evolutionary relationship of Lauraceae, especially in the genus *Lindera*.

## 2. Materials & Methods

### 2.1. Sample Collection, DNA Extraction and Sequencing

The samples of *L. aggregata* (Wu-Yao) in this experiment were provided by the Zhejiang Hongshiliang Group Tiantaishan Wuyao Co. (Taizhou, China). Fresh leaves were collected, quick-frozen with liquid nitrogen and brought back to the laboratory for extraction of total genomic DNA using the modified CTAB technique [30]. The extracted DNA samples were preliminarily examined with a 1% agarose gel, and NanoDrop was used to test the concentration and purity of the extracted DNA. Qualified DNA samples were sent to Novogene Biotechnology Co., Ltd. (Beijing, China). for double-end sequencing via the Illumina HiSeq 6000 platform with a sequence read length of 150 bp. The raw reads obtained were preprocessed with Trimmomatic v0.39 software [31], including removing the reads with adapters, with a proportion of N greater than 10% and of low-quality. The clean reads were then evaluated for treatment using FastQC v0.11.7 software. Moreover, the chloroplast genomes of samples related to *L. aggregata* were downloaded from the NCBI database for subsequent comparative analyses, including 26 species of the *Lindera* genus and 9 species of different genera in the Lauraceae family. The detailed information was recorded in Table S1.

### 2.2. Assembly and Annotation of the Chloroplast Genomes

First, the complete chloroplast genome sequence of *L. aggregata* was assembled with Getorganelle v1.7.7 [32] software based on clean reads using the chloroplast genome of *L. glauca* as the reference sequence. Then, CPGAVAS2 [33] and GeSeq [34] online databases were utilized to obtain the annotation information of the assembled chloroplast genome, and the annotation results were imported into Geneious Prime v11.0.18 [35] software for manual correction to obtain accurate annotation information. Finally, CHLOROPLOT [36] online tool (https://irscope.shinyapps.io/Chloroplot/ (accessed on 20 January 2024)) was used to visualize the mapping of the chloroplast genome and submit the genome sequence to the NCBI database to obtain the accession number. Furthermore, due to the large amount of unverified information in the chloroplast genomes of the downloaded species, the chloroplast genomes were also re-annotated with the same method described above in order to carry out subsequent comparative analyses.

### 2.3. Characteristics Analysis of Chloroplast Genomes

The length of the total chloroplast genome sequence, LSCs, SSCs and IRs, and GC content of the chloroplast genomes of each species were obtained using the CPGView [37] online tool (http://47.96.249.172:16085/cpgview/home (accessed on 20 January 2024)), and the number of protein-coding genes (PCGs), tRNAs, and rRNAs were counted.

Relative synonymous codon usage (RSCU) values were calculated for PCGs in the chloroplast genomes using CodonW v1.4.2 software. Three stop codons (TAA, TAG and TGA) without degeneracy were deleted prior to analysis. An RSCU value > 1 represents a preference for the use of the codon, RSCU = 1 indicates that there is no usage preference for the codon and RSCU < 1 indicates that the codon is used less frequently. This codon preference is considered to be the result of a combination of natural selection, species mutation and genetic drift [38].

The simple sequence repeats (SSRs) in the chloroplast genomes were identified using the microsatellite online identification tool MISA-web [39]. The following parameter settings were used: the minimum number of repeats for the mononucleotide (Mono-) repeats, dinucleotide (Di-) repeats, trinucleotide (Tri-) repeats, tetranucleotide (Tetra-) repeats, pentanucleotide (Penta-) repeats and hexanucleotide (Hexa-) repeats were set to 10, 5, 4, 3, 3 and 3, respectively. Furthermore, to detect forward repeats (F-), reverse repeats (R-), palindromic repeats (P-) and complementary repeats (C-) in the chloroplast genome sequences, we searched the chloroplast genomes of all species using the online tool REPuter (https://bibiserv.cebitec.uni-bielefeld.de/reputer/ (accessed on 20 January 2024)) [40] with the parameters set as follows: minimum repeat length greater than 30 bp, maximum repeat length of 300 bp and Hamming distance of 3.

### 2.4. Comparative Genomics Analysis of the Chloroplast Genome

Diversity assessment of the chloroplast genomes of species among different genera of Lauraceae and within the genus *Lindera* was carried out using the mVISTA [41] online tool (https://genome.lbl.gov/vista/mvista/submit.shtml (accessed on 20 January 2024)), with the mode selection of shuffle-LAGAN. *L. aggregata* was used as a reference sequence to elucidate the level of sequence divergence.

PhyloSuit v1.2.3 software [42] was used to extract PCGs from the chloroplast genomes. The extracted PCGs were first compared using MAFFT v7.427 software [43]. Then, the KaKs_Calculator 2.0 tool [44] was used to calculate the ratio of nonsynonymous (Ka) to synonymous (Ks) substitutions (Ka/Ks), with the default model (MLWL).

The IRScope online program [45] was used to visualize the connectivity sites of the regions and to detect the expansion and contraction of the chloroplast genome boundary regions between IRs, SSCs and LSCs.

The Mauve v2.4.0 program in the Geneious Prime software was used for rearrangement and structural comparison of chloroplast genomes to analyze the presence of missing genes, duplicates, rearrangements or translocations, which can probe the structural variability characterizing the chloroplast genomes of Lauraceae plants. Moreover, nucleotide diversity (Pi) values were calculated using the DnaSP v6.12.03 software [46], with a sliding window length and step size of 600 bp and 200 bp, respectively.

### 2.5. Phylogenetic Analysis

A phylogenetic tree was constructed based on the sequenced and assembled chloroplast genomes of *L. aggregata* in this study, as well as all the published chloroplast genomes of the *Lindera* genus on NCBI (total of 26) and 12 chloroplast genomes of different genera of Lauraceae, with *C. camphora*, *C. parthenoxylon*, *C. glanduliferum* and *C. insularimontanum* as outgroups. The chloroplast genome information was detailed in Table S1.

Firstly, the chloroplast genome sequences of all species were imported into PhyloSuit v1.2.3 software to extract the common PCGs. Then the MAFFT module was used to perform multiple sequences alignment and trimmed using the trimAl v1.2 software [47]. Then, the aligned and trimmed sequences were concatenated via a concatenate module to obtain a dataset of 39 concatenated Lauraceae common PCGs. Finally, phylogenetic tree construction was performed using the maximum likelihood (ML) method and Bayesian inference (BI) method, respectively. The ML trees were constructed using IQ-TREE v2.2.0.3 software [48], 5000 ultra-fast bootstraps were set up and the optimal model was obtained via the ModelFinder program [49]. The BI tree was constructed using MrBayes v3.2.7a software [50], with the model selected as GTR+I. MCMC was run for 5 million generations, with sampling every 1000 generations, with the top 25% of the trees aged and discarded and the rest constructed as a 50% consistent tree; convergence was determined by keeping the average standard deviation of the crossover frequencies < 0.01. Finally, the topologies of the phylogenetic trees were visualized using the Chiplot online tool [51].

### 2.6. Divergence Time Estimated

The divergence time within the genus *Lindera* of Lauraceae was estimated with BEAST v2.7.6 software [52] and with reference to previous studies [53,54]; the similar species (*Liriodendron chinense* and *Piper nigrum*) of different families were taken as outgroups. The divergence time between Lauraceae and Magnoliaceae (approximately 124 Mya) and Magnoliaceae and Piperaceae (approximately 142.8 Mya) were found through the Timetree (http://www.timetree.org/ (accessed on 20 January 2024)) online database, and these were used as the time correction point of the evolutionary tree. The evolutionary tree was constructed with a GTR model and strict clock. The total operating generation was set to 10,000,000, and sampling was carried out for every 1000 generations. After operation, the stability of the results was evaluated with Tracer v1.7.2 software [55]; the ESS values were all more than 200, indicating that the results converged and the evolutionary tree was reliable. Using TreeAnnotator v2.6.7 software, after burning 50% of the total generations, a phylogenetic tree containing the time of species differentiation was generated and visualized using Chiplot online tool (https://www.chiplot.online/ (accessed on 20 January 2024)) [51].

## 3. Results

### 3.1. Analysis of Characteristics of Chloroplast Genomes

**Figure 1:**
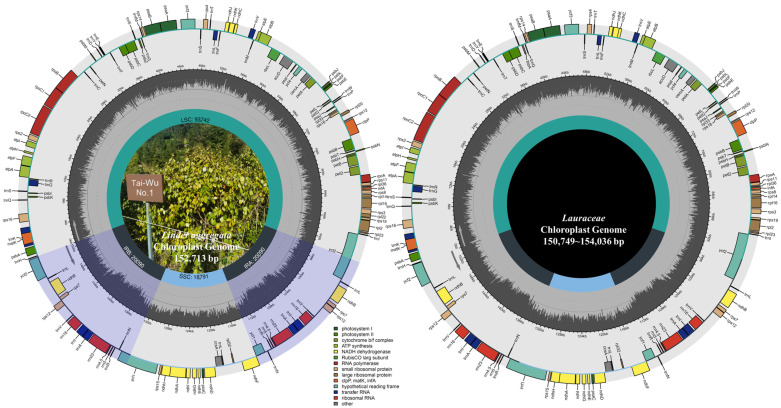
Chloroplast genome structure map of *L. aggregata* to the left and 35 Lauraceae species. The genes inside and outside the circle are transcribed clockwise and counterclockwise, respectively. Genes with different functions are given different colors. The gray in the inner circle represents the GC content and shows the corresponding regions and corresponding lengths of the four blocks (LSC/SSC/IRa/IRb).

The chloroplast sequence of *L. aggregata* was preliminarily annotated after assembly, and manually corrected with the most similar sequences as references (Figure 1). At the same time, eight unverified chloroplast genomes (Table S1) in the NCBI database were re-annotated, and the remaining sequences were uniformly corrected to avoid differences in sequence annotation caused by humans. In order to compare the differences between the plants of the same genus and the plants of different genera in Lauraceae, 36 plants were divided into two groups for comparative analysis. Within the *Lindera* genus, the length of chloroplast genomes ranged from 152,478 bp (*L. benzoin*) to 153,679 bp (*L. pulcherrima* var. *attenuata*), LSC length ranged from 93,145 to 93,921 bp, the length of SSC was 18,791–19,800 bp, the length of IRs was 20,047–20,474 bp and the total GC content was 39.1–39.2%. Among the genera, the length of chloroplast genomes ranged from 150,749 bp (*Nectandra angustifolia*) to 154,036 bp (*Neolitsea confertfolia*), the length of LSC was 93,381–93,795 bp and the length of SSC was 18,382–18,969 bp; IRs length was 19,292–20,781 bp, and the total GC content was the same as above (Table S2). In general, the chloroplast genomes of all species showed typical quadripartite structures (Figure 1), with little change in overall GC content. Furthermore, whereas overall and IRs length differences were greater between genera than within genera, LSC and SSC differences were greater within genera than among genera.

The majority of Lauraceae species contained 112 unique genes, 78 protein coding genes (PCGs), 30 tRNA genes and 4 rRNA genes (Table S2). Whereas a few species (*L. glauca*, *L. erythrocarpa*, *L. latifolia*, etc.) lacked trnG-GCC genes (Table S3). Moreover, *Cinnamomum glanduliferum* and *N. angustifolia* had one extra *ycf68* gene. All these genes were divided into four categories after functional annotation: photosynthesis, self-replication, other genes and unknown function genes. Among these genes, nine PCGs (*ndhA*, *ndhB*, *petB*, *petD*, *atpF*, *rpl16*, *rpl2*, *rps16* and *rpoC1*) and six tRNA genes (*trnA-UGC*, *trnG-UCC*, *trnI-GAU*, *trnK-UUU*, *trnL-UAA* and *trnV-UAC*) had one intron, whereas *clpP*, *rps12* and *ycf3* had two introns. Moreover, 14 genes (*ndhB*, *rps12*, *rps7*, *rrn16S*, *rrn23S*, *rrn4.5S*, *rrn5S*, *trnA-UGC*, *trnI-GAU*, *trnL-CAA*, *trnN-GUU*, *trnR-ACG*, *trnV-GAC*, *ycf1* and *ycf2*) had two copies, and one copy of *ycf1* and *ycf2* genes, as well as *rpl2,* were pseudogenes (Table S3).

### 3.2. Expansion/Contraction Analysis of the Boundary

**Figure 2:**
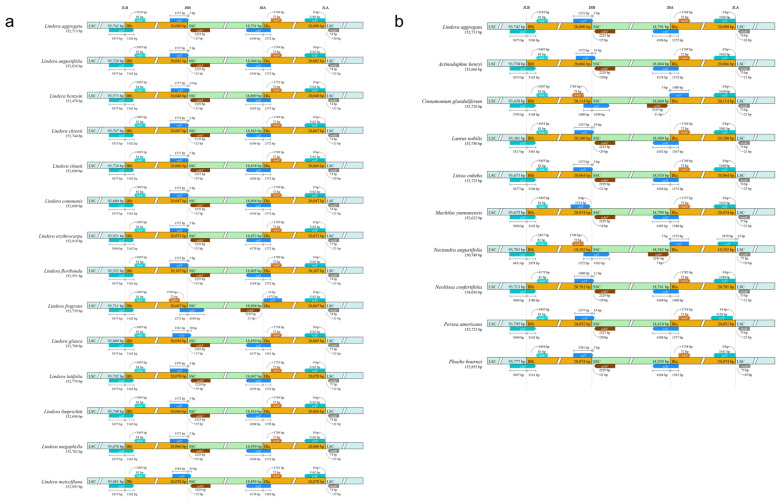
Comparison of junction boundaries in four regions (LSC/SSC/IRa/IRb) between *L. aggregata* and species of the same genus (**a**) and different genera (**b**). The number on the color gene indicates the distance between the gene and the edge of the border. Some details of junction boundaries between *L. aggregata* and species from the same genus are shown in Figure S1.

In order to detect the differences of chloroplast genome sequences between *L. aggregata* and species of the same genus and different genera, we used IRscope v0.1 software to visualize their partition length and boundary information (Figure 2 and Figure S1). The results showed that the structure and size of chloroplast genomes were highly conservative between genera and within genera, but the IR boundaries were different among species due to the contraction/expansion of IRs/SCs. Within the genus, the coding region of the *ycf1* gene was crossed by the boundary between IRs and the SSC. The 4175–4204 bp region of *ycf1* was located in the SSC region, whereas another shorter copy of the *ycf1* gene passed through the IRs, and the 4–65 bp region was located in the SSC region. The longer *ycf2* copy gene was located in the boundary region between the LSC and IRb, and the 3267–3675 bp region was located in the LSC region (Figure 2a and Figure S1). Among genera, the results were similar to those within the genus. The longer *ycf1* and *ycf2* genes were located at the boundary of the IR/SC, and the regions of 4160–4196 bp and 3317–4431 bp were located in the SSC region and LSC region (Figure 2b), respectively. In general, the expansion/contraction of IRs led to changes in the length of the whole chloroplast genomes and changes in the boundary position between the LSC and SSC.

### 3.3. Comparative Chloroplast Genome Sequences Differences

**Figure 3:**
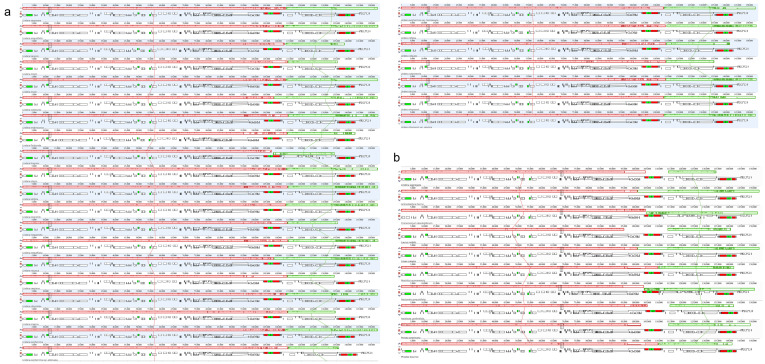
MAUVE alignment of chloroplast genomes between *L. aggregata* and species of the same genus (**a**) and species of different genera (**b**). *L. aggregata* is shown at the top as a reference genome. In each alignment, local collinear blocks are represented by areas of the same color connected by lines.

Taking the chloroplast genome of *L. aggregata* as the reference sequence to compare the differences of chloroplast genome sequences between intra-genus (Figure S2) and inter-genera (Figure S3), within the genus, 26 chloroplast genomes can be divided into two groups of sequences with high similarity. One group includes *L. praecox*, *L. pulcherrima* var. *hemsleyana* and *L. rubronervia*; the variations of the sequences were mainly in the three regions of *trrH-GUG*, *psbA* and *ccsA-ndhD*, but the coding regions were still more conservative than the noncoding regions. The overall variation of the other group of sequences (Pi = 0.00365) was smaller than the above group (Pi = 0.00845), and both the coding region and the noncoding region were highly similar. Among different genera, nine chloroplast genomes were somewhat similar as a whole, but there were great differences between *Actinodaphne henryi*, *N. angustifolia* and *Laurus nobilis* and other species in the non-coding region (Figure S3). Furthermore, we compared 36 intra-genus and inter-genera chloroplast genomes; the results showed that there were local collinear regions between intra-genus (Figure 3a) and inter-genera (Figure 3b) chloroplast genomes, all genes were highly consistent and there was no gene rearrangement and inversion.

### 3.4. Analysis of a Highly Variable Region of Chloroplast Genomes

**Figure 4:**
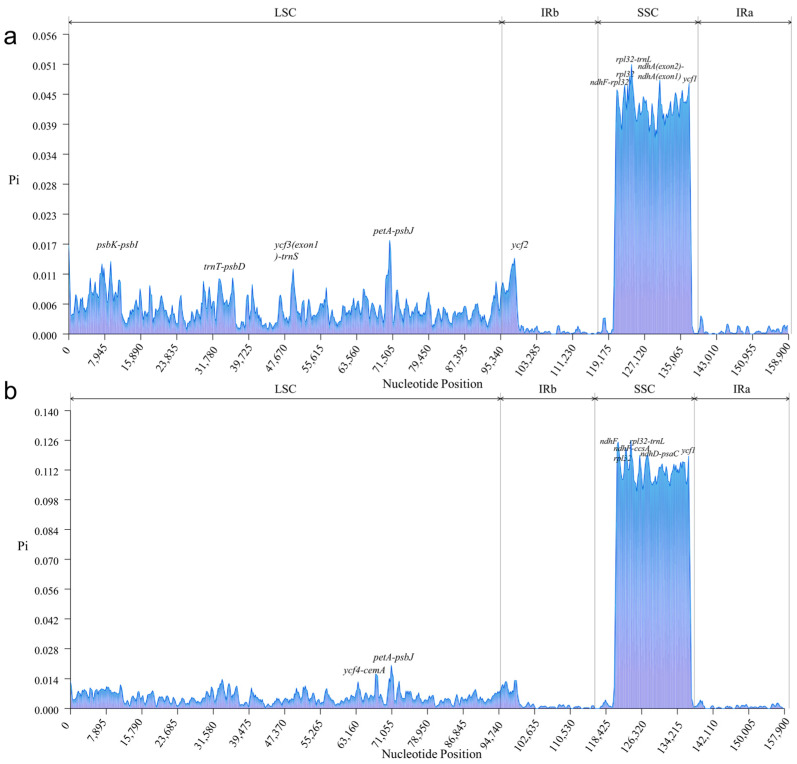
Comparison of nucleotide diversity (Pi) values of chloroplast genomes. (**a**) Comparison of Pi values between *L. aggregata* and 26 species of the same genus. (**b**) Comparison of Pi values between *L. aggregata* and 9 different genera of Lauraceae. *X*-axis: positions of the midpoints of a window, *Y*-axis: nucleotide diversity in each 600 bp window.

In order to detect the differences of chloroplast genome sequences within genus and among different genera, we calculated the nucleotide diversity (Pi) between *L. aggregata* and 26 plants of *Lindera*, *L. aggregata* and nine different genera of Lauraceae. The Pi values of 27 genomes within the genus ranged from 0 to 0.05068 (average = 0.00787), and the Pi values of 10 genomes among different genera ranged from 0 to 0.12565 (average = 0.01548). Moreover, five hypervariable loci were identified within genus (Figure 4a), namely *ycf1* (0.04708), *ndhA*(exon2)-*ndhA*(exon1) (0.04772), *rpl32-trnL* (0.05068), *rpl32* (0.04677) and *ndhF-rpl32* (0.04684). Six hypervariable loci were identified among genera (Figure 4b), namely *ycf1* (0.11833), *ndhD-psaC* (0.11894), *rpl32-trnL* (0.12565), *ndhF* (0.12495), *ndhF-rpl32* (0.12194) and *ccsA* (0.1187). They were all located in the SSC region, indicating that the SSC region was much more different than the IR and LSC regions, and the IR region was highly conservative, which was consistent with the above analysis.

### 3.5. SSRs and Interspersed Repeat Sequence Analysis

**Figure 5:**
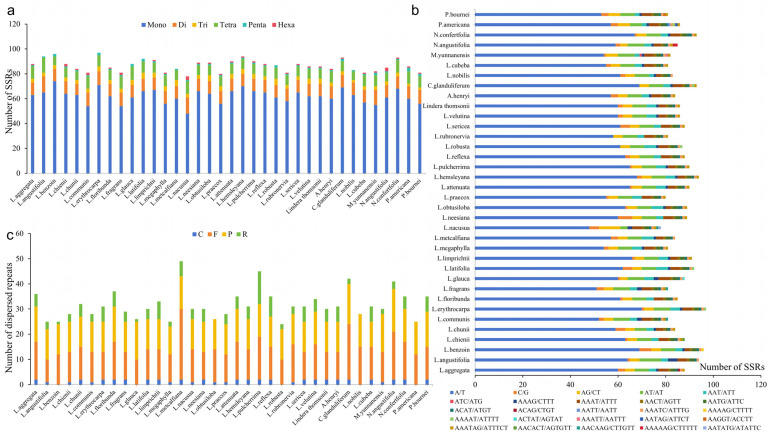
Comparison of SSRs and interspersed repeat sequences across 36 Lauraceae chloroplast genomes. (**a**) The number of the different SSRs types. (**b**) The number of SSR motifs in different repeat types. (**c**) The number of different interspersed repeat types.

Simple sequence repeats (SSRs) were widely distributed in chloroplast genomes. There were 78–97 SSRs detected across each of chloroplast genomes from 36 Lauraceae plants, for a total of 3124 SSRs. SSR types ranged from mononucleotide (Mono-) repeats to hexanucleotide (Hexa-) repeats, although not all six types of SSRs existed in each taxon (Figure 5a,b). Mono-repeat sequences were the most common (48–74 SSRs), which were mainly composed of A/T type, and there were only 0–6 G/C repeats. Dinucleotide (Di-) repeats were composed of AG/CT and AT/AT; the number of each was equal, both representing 3–9 SSRs. Except that the trinucleotide (Tri-) repeats of *Machilus yunnanensis* were composed of AAT/ATT and ATC/ATG types, all the other species had only AAT/ATT type with a number of 2–5. There were 7–12 tetranucleotide (Tetra-) repeats, including seven types, but ACAG/CTGT and AATT/AATT types only existed in *L. aggregata* and *P. americana*, respectively. There were 1–3 pentanucleotide (Penta-) repeats, including seven types. Except that the AAATC/ATTT repeat existed in all species, the other six types existed in different species. There were 0–3 Hexa- repeats, including five types, of which the AACACT/AGTGTT type existed only in *L. chunii*, the AAAAAG/CTTTTT type only existed in *N. angustifolia*, with two repeats, and the AATATG/ATATTC type only existed in *L. nacusua*.

In addition, we also detected interspersed repeat sequences (24–49) in these chloroplast genomes, for a total of 1142 interspersed repeat sequences, including four repeat types: forward repeats (F-), reverse repeats (R-), palindromic repeats (P-) and complementary repeats (C-). P-repeats and F-repeats appeared most frequently, being detected 457 and 481 times, respectively; R- repeats and C- repeats appeared less frequently, being detected 151 and 53 times, respectively. R-repeats and C-repeats were not even detected in all species; no C-repeats were detected in *L. benzoin* and *L. glauca* and *L. robusta*; no R-repeats were detected in *L. obtusiloba*, and neither of them was detected in *L. nobilis* and *P. americana*.

### 3.6. Analysis of Codon Bias and Selection Pressure in Chloroplast Genomes

**Figure 6:**
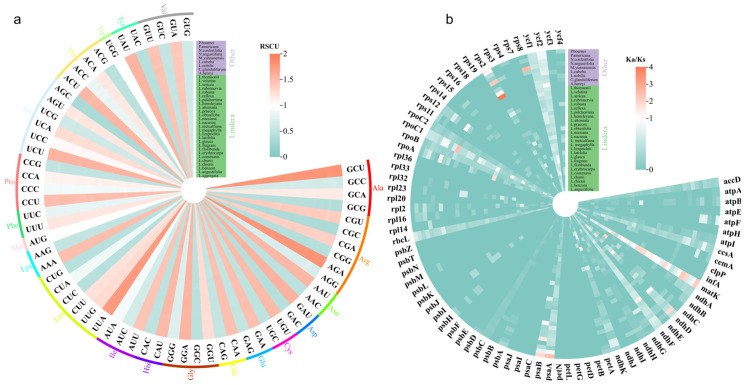
Codon bias analysis of chloroplast genomes (**a**) and selection pressure analysis of protein coding genes (**b**) in 36 Lauraceae species. The amino acids corresponding to different codons in a graph are represented by arcs of different colors. The species of the same genus of *Lindera* and different genera of Lauraceae are distinguished by purple and green backgrounds.

Codon bias analysis of 36 chloroplast genomes of Lauraceae showed that 61 codons were detected, encoding 20 amino acids (Figure 6a). The most common ones were arginine (Arg), leucine (Leu) and serine (Ser), using six codons, then alanine (Ala), glycine (Gly), proline (Pro), threonine (Thr) and valine (Val), using four codons. The least common were tryptophan (Trp) and methionine (Met), which used one codon. Based on the computational relative synonymous codon usage (RSCU), 30 codons were greater than 1, which indicated that these codons were biased in the chloroplast genome, and most of them ended with A or U; 2 codons (AUG and UGG) were equal to 1, indicating that the two codons had no preference; 29 codons were less than 1, indicating that these codons were used less frequently, and most of them ended in G or C. In these chloroplast genomes, the AGA codon encoding Arg was the most commonly used, with RSCU values of 1.77–1.82, and the AGC codon encoding Ser exhibited the lowest frequency, with RSCU values that ranged from 0.33 to 0.37. The results showed that the majority of codons in the chloroplast genome of Lauraceae were biased.

Taking the chloroplast genome of *L. aggregata* as the reference sequence, the selection pressures of genes from Lauraceae were evaluated by calculating Ka/Ks values (Figure 6b). The results showed that the Ka/Ks values of most of the 79 PCGs in all species were less than 1 or could not be determined because the value of Ka or Ks was zero, which also indicated that they were conservative. Furthermore, the Ka/Ks values of some genes showed differences among different species, such as the *matK* and *rpoC1* genes of *L. glauca* and *L. angustifolia*; the *matK* gene of *L. chienii*, *L. benzoin*, *L. limprichtii*, *L. erythrocarpa*, *L. metcalfiana*, *L. neesiana*, *L. attenuata*, *L. hemsleyana*, *L. pulcherrima*, *L. reflexa*, *L. robusta*, *L. rubronervia*, *L. sericea* and *L. thomsonii*; the *ndhD* gene of *L. communis*, *L. fragrans* and *P. americana*; the *rpoC1* gene of *L. megaphylla*; the *matK* and *ndhD* genes of *L. nacusua*; the *rpoA* gene of *L.obtusiloba* and *L. cubeba*; the *matK, ndhD, ndhF, psaB* and *rpoC1* genes of *A. henryi*; the *rps2* gene of *C. glanduliferum* and *L. nobilis*; the *matK* and *psaB* genes of *M. yunnanensis*; the matK, *rps2* and *rps8* genes of *N. angustifolia* and the *matK*, *rps2, psaA* and *psbB* genes of *P. bournei*. The Ka/Ks values of these genes were greater than 1, and the values among different genera were generally greater than those within the genus. The above results showed that all genes except 10 genes (*matK, rpoA, rpoC1, ndhB, ndhD, ndhF, psaA, psaB, rps2* and *rps8*) of individual species were purifying selections.

### 3.7. Phylogenetic Trees and Divergence Time Analysis of the Chloroplast Genomes

**Figure 7:**
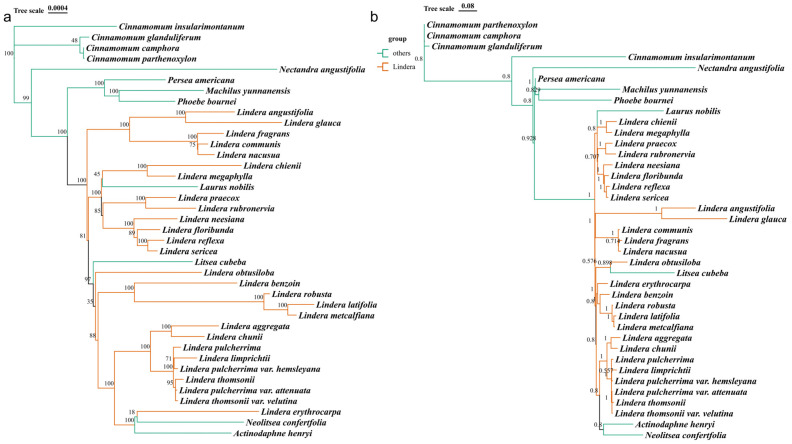
The maximum likelihood (ML) method in (**a**) and Bayesian inference (BI) method in (**b**) for generating phylogenetic trees based on the common protein coding genes from 39 Lauraceae plants. The support rate values were shown on the branches of the trees.

In this study, based on the PCGs of chloroplast genomes of 27 *Lindera* species and 12 Lauraceae species, phylogenetic trees were constructed with maximum likelihood (ML) and Bayesian inference (BI) methods, respectively. The results showed that the two phylogenetic trees had similar topological structure, but the support rate obtained with the BI method was generally higher (Figure 7b), whereas the topological relationship of some terminal branches of the ML method was difficult to distinguish (Figure 7a). From the topological structure of the phylogenetic tree, it could be seen that all the plants of *Lindera* and Wuyao were clustered into one big branch, but they were also mixed with other genera of Lauraceae (*Actinodaphne*, *Laurus*, *Litsea* and *Neolitsea*), whereas the remaining different genera (*Cinnamomum*, *Nectandra*, *Persea*, *Phoebe* and *Machilus*) were clustered into another single big branch, which was obviously different from *Lindera*.

**Figure 8:**
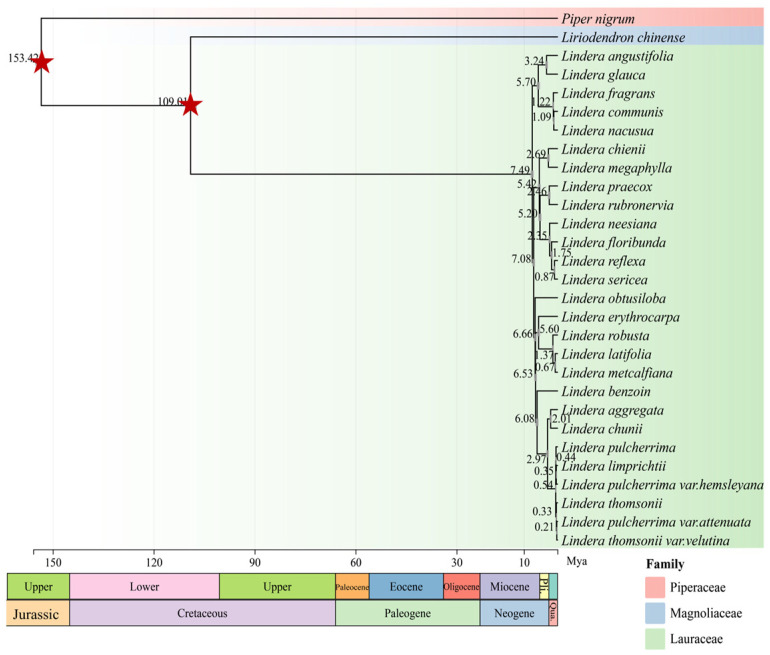
An evolution tree with divergence time. The grey bar chart on the evolutionary branch represents the 95% HPD boundary, and the number represents the average of differentiation time. The two red, five-pointed stars represent the time of the fossil calibration. Different colors are used to represent different geological periods under the evolutionary tree.

Furthermore, based on the chloroplast genomes data, taking *L. chinense* (Magnoliaceae family) and *P. nigrum* (Piperaceae family) as outgroups, the divergence time of *Lindera* (Lauraceae family) was obtained, and the average estimated age and 95% HPD interval were mapped to the geological periods (Figure 8). The crown age of Lauraceae was estimated to be 109.01 Mya (95% HPD: 107.18–110.74 Mya) in the Lower Cretaceous epoch. From the late Miocene to the Pleistocene, the species of the genus *Lindera* experienced rapid diversification. *L. aggregata* and *L. chiunii* formed after differentiation in 2.01 Mya (95% HPD: 1.69–2.35 Mya). *L. pulcherrima* and its two varieties (*L. pulcherrima* var. *attenuata* and *L. pulcherrima* var. *hemsleyana*), *L. limprichtii* and *L. thomsonii* and one of its varieties (*L. thomsonii* var. *velutina*) were closely related to each other, and their internal differentiation was the latest to occur (0.21–0.54 Mya).

## 4. Discussion

We used the second generation high-throughput sequencing technique to sequence “Tiantai Wu-Yao” and obtained a complete chloroplast genome via assembly and annotation and re-annotated eight uncertified chloroplast genomes in the same genus to correct errors (Table S1). The chloroplast genome of *L. aggregata* was the same as that of most angiosperms, with conservative circular quadripartite structures, namely two IR regions, one LSC region and one SSC region [56,57,58]. The total length of the sequence was 152,713 bp, which occupied a middle position between the same genus (152,478–153,679 bp) and different genera (150,749–154,036 bp). The content of GC was 39.2%, which was basically no different from other plants of the genus *Lindera* and was similar to that of different genera of Lauraceae [18,19,23]. The total number of chloroplast genes was 125, which was basically no different from plants in the same genus. The differences from plants in different genera mainly came from PCGs and tRNA genes, whereas rRNAs genes were the most conserved, with eight genes in all species. Gene loss often occurred during the evolution of the chloroplast genome in angiosperms, especially the contraction/expansion of IR regions which lead to gene loss/increase [59]. Through comparative analysis, it was found that only three taxa (*L. erythrocarpa*, *L. glauca* and *L. latifolia*) of the same genus and nine taxa of different genera lacked the *trnG-GCC* gene, whereas *C. glanduliferum* and *N. angustifolia* had one more *ycf68* gene. It was reported that there were two kinds of gene rearrangements in the *ycf68* gene, which may be a pseudogene [60,61].

Chloroplast genomes of higher plants usually show boundary contraction/expansion differences in different genera or even within the same genus, which is also the main factor leading to changes in gene length and number in different species [62,63]. It was amazing that between species of the same genus or different genera, except for the fact that the *trnL* genes of *L. sericea* and *L. reflexa* were located in the LSC region due to the expansion of the IRb region, *trnL*, *ndhF* and *trnN* were consistently, respectively, located in the corresponding boundary region of IR/SC. Moreover, the expansion of the IR region also caused genes with multiple copies to produce pseudogenes (*ycf1* and *ycf2*) in the boundary region of IRa/SSC and IRb/LSC. Similar to the results of most studies, pseudogenes were easy to be produced in the chloroplast genome of the boundary region of SC/IR in most terrestrial plants [18,63].

The whole chloroplast genome sequence of *L. aggregata* was compared with species of the same genus and with species of different genera. The results showed that Lauraceae plants were highly conservative as a whole and there was no gene rearrangements or inversions. The variation degree of the LSC and SSC region were higher than that of the IR region, and that of the non-coding region was higher than that of the coding region, which was similar to the majority of higher plants [24,63,64]. Furthermore, five highly variable regions (*ycf1*, *ndhA*(exon2)-*ndhA*(exon1), *rpl32-trnL*, *rpl32* and *ndhF-rpl32*), six highly variable regions (*ycf1*, *ndhD-psaC*, *rpl32-trnL*, *ndhF*, *ndhF-rpl32* and *ccsA*) and three common regions (*ycf1*, *rpl32-trnL* and *ndhF-rpl32*) were found within and between genera via sliding windows analysis of nucleotide diversity detection, which were mainly located in intergenic regions. Among them, *rpl32-trnL* had the highest degree of variation within genus and between genera (Pi = 0.05068 and 0.12565), and there were many variation sites. Similar results had been found in the genera *Alseodaphne*, *Alseodaphnopsis* and *Dehaasia* of the Lauraceae family [24,65,66,67]. Therefore, *rpl32-trnL* can be used as an important DNA marker within the genus *Lindera* or among different genera of Lauraceae.

Repetitive sequences affect gene transcriptional regulation, protein translation, chromosome formation and metabolism and reflect the differences in mutation frequency and the evolution rate of species [68]. There were 24–49 interspersed repeat sequences in the chloroplast genomes of Lauraceae, among which *L. benzoin*, *L. robusta* and *L. glauca* lacked the C- repeat type, *L. obtusiloba* lacked the R- repeat type, *L. nobilis* and *P. americana* only contained F- and P- repeat types and other species contained F-, R-, P- and C- repeat types. Moreover, the SSRs loci are widely distributed in the genome of organisms, which can lead to diversity due to the different number of repetitive units [69]. At the same time, it has the characteristics of genetic stability, high abundance and wide distribution, so it is often used for species identification and genetic diversity detection [70]. In this study, 78–97 SSR loci were found in Lauraceae, including six repetitive types. Among them, the mono- repeat sequence was the most common, accounting for 61.5–77.1% of the total SSRs, with the A/T base as the basic repeat unit.

The selection pressures of the genes are detected by calculating the Ka/Ks, in which, when Ka/Ks > 1, the genes are in the positive selection state, when Ka/Ks = 1, the genes are in the neutral evolution state and when Ka/Ks < 1, the genes are in the purified selection state [63,71]. The average Ka/Ks values of Lauraceae (0.1230–0.3282) were all less than 1, of which 79 PCGs’ Ka/Ks values were less than 1 in all species, indicating that the majority of genes were in the state of purifying selection. Only in some individuals there were 10 genes (*matK*, *rpoA*, *rpoC1*, *ndhB*, *ndhD*, *ndhF*, *psaA*, *psaB*, *rps2* and *rps8*) associated with maturase, photosystem I, NADPH dehydrogenase, RNA polymerase and small ribosomal proteins, and their Ka/Ks were greater than 1, indicating that these genes were affected by positive selection. They were undergoing a period of rapid evolution. Furthermore, codon usage bias can reflect the evolution characteristics of the chloroplast genome and its influencing factors and plays an important role in genome expression [72]. The RSCU value reflects the codon usage patterns of different genes, and the higher the value, the higher the codon usage frequency [38]. In Lauralee, the RSCU values of 30 codons were greater than 1, and 97% of these high-frequency codons end with A/U, which was consistent with the codon usage bias of chloroplast genes in other plants [73], indicating that there was asymmetry in the third base of chloroplast codons and a bias of A/U.

The taxonomic status of Lauraceae has always been controversial in the academic community. Some traits used in morphological classification are affected by environmental and geographical factors, so it is difficult to establish an accurate phylogenetic relationship. The chloroplast genome is highly conservative and widely used in phylogenetic research. Song et al. constructed a phylogenetic tree based on the chloroplast genome of eight species from Lauraceae, which revealed that *Persea*, *Machilus* and *Phoebe* were closely related [25], which was consistent with the conclusion of Rohwer et al. which was based on an ITS sequence [65] and Li et al. which was based on ITS and LEAFY intron II sequences [67]. Zhao et al. constructed a systematic tree containing 32 species of Lauraceae. The results showed that the family branches of *Cinnamomum*-*Ocotea* included *Sassafras*, *Cinnamomum*, *Ocotea* and *Nectandra*, and this branch formed a sister group with the branches of *Lindera* (including *Lindera*, *Laurus* and *Litsea*) [22]. Tian et al. reconstructed the phylogenetic tree containing 49 species of Lauraceae based on the chloroplast genome, which supported the phylogeny of *Laureae*, *Perseae* and *Cinnamoneae*, and found that *Linera*, *Litsea* and *Iteadaphne* were closely related [20]. Liu et al. reconstructed the phylogeny of Lauraceae using 191 chloroplast genes (25 genera and 131 species). The results showed that Lauraceae could be divided into seven sections: sect. Cryptocaryeae, sect. Cassytheae, sect. Neocinnamomeae, sect. Caryodaphnopsideae, sect. Perseae, sect. Cinnamoneae and sect. Laureae [74]. The application of the chloroplast genome has solved a majority of the problems in the phylogenetic status of Lauraceae and has high reference value. In this study, the phylogenetic relationship within *Lindera* and among different genera of Lauraceae was studied with *L. aggregata* as the center. The results showed that most genera of Lauraceae could be distinguished from *Lindera*, but some species of *Lindera*, *Litsea* and *Laurus* formed their own subgroups, which was consistent with the above results. Moreover, it was interesting that *L. aggregata* and some plants of the same genus form a subgroup, which was closely related to the genera *Neolitsea* and *Actinodaphne*. Finally, through the evaluation of the divergence time, it was inferred that the crown age of Lauraceae was 109.01 Mya (95% HPD: 107.18–110.74 Mya) in the Lower Cretaceous epoch. The above divergence time was much younger than the divergence time of genome assessment (average: 119.8 Mya, HPD: 101.9–135.6 Mya) [53], which may be caused by a chloroplast capture event. Moreover, from the late Miocene to the Pleistocene, the species of the genus *Lindera* experienced rapid diversification. *L. aggregata* and *L. chiunii* were formed after differentiation in 2.01 Mya (95% HPD: 1.69–2.35 Mya).

## 5. Conclusions

In this study, we sequenced, assembled and annotated the chloroplast genome of *L. aggregata* (Wu-Yao) and re-annotated a large number of unverified chloroplast genomes of Lauraceae. For the first time, we compared the structure and differences of chloroplast genomes within genus and between genera in detail. These chloroplast genomes were highly conserved, with a total of 111–112 unique genes in each genome, including 78–79 PCGs, 29–30 tRNA and 4 rRNA genes. Moreover, the boundary and collinear analysis of genomes also showed that these genomes were highly conservative and there was no gene rearrangement or inversion. Among these genomes, there were six types of SSRs and four types of interspersed repeats, among which the Mono-repeat sequences had the highest frequency of SSR types, and a majority of them toke A/T as the basic repeat unit; F- and P-repeats were the main types of interspersed repeats. Codon bias analysis showed that different species had similar RSCU values, and all tended to end with A or U bases. Five highly variable regions were identified within the genus, namely *ycf1*, *ndhA*(exon2)-*ndhA*(exon1), *rpl32-trnL*, *rpl32* and *ndhF-rpl32*; there were six highly variable regions among genera, namely *ycf1*, *ndhD-psaC*, *rpl32-trnL*, *ndhF*, *ndhF-rpl32* and *ccsA*; and there were three highly variable regions (*ycf1*, *rpl32-trnL* and *ndhF-rpl32*) shared by them. These regions can be used as vital markers for classification and identification within and between genera. Selective pressure analysis showed that a majority of genes were under purifying selection, the sequences were relatively conservative and only 10 genes (*matK*, *rpoA*, *rpoC1*, *ndhB*, *ndhD*, *ndhF*, *psaA*, *psaB*, *rps2* and *rps8*) were positively selected in some individuals. The evolutionary trees clarified the phylogenetic relationship of Lauraceae and revealed the taxonomic status of *Lindera*. The estimation of the divergence time revealed that the divergence time of *Lindera* (Lauraceae) was in the Lower Cretaceous epoch. After that, they experienced rapid adaptive radiation from Miocene to Pleistocene, and Wu-Yao was formed after differentiation in the middle of early Pleistocene.

## Data Availability

The newly assembled complete chloroplast genomic sequences of *Lindera aggregata* can be obtained on GenBank, and the accession number is PP199190.

## Supplementary Materials

The following supporting information can be downloaded at: https://www.mdpi.com/article/10.3390/genes15030263/s1, Figure S1: Comparison of junction boundaries in four regions (LSC/SSC/IRa/IRb) of 13 chloroplast genomes. The number on the color gene indicates the distance between the gene and the edge of the border. JLB represents LSC/IRb boundary, JSB represents SSC/IRb boundary, JSA represents SSC/IRa boundary, and JLA represents LSC/IRa boundary; Figure S2: The sequence difference map compares 26 chloroplast genomes of *Lindera*, using the chloroplast genome of *L. aggregata* as the reference sequence via mVISTA software. The gray arrows and thick black lines above indicate the direction of the gene. Different areas were given different colors. The pink region was a conservative non-coding region (CNS), purple was the gene exon region, and the blue region was tRNA or rRNA; Figure S3: The sequence difference map compares 9 chloroplast genomes of Lauraceae, using the chloroplast genome of *L. aggregata* as the reference sequence via mVISTA software. The gray arrows and thick black lines above indicate the direction of the gene. Different areas were given different colors. The pink region was a conservative non-coding region (CNS), purple was the gene exon region, and the blue region was tRNA or rRNA; Table S1: Details of chloroplast genomes for 39 species; Table S2: Characteristics of 36 chloroplast genomes in Lauraceae; Table S3: Gene annotation of Lauraceae chloroplast genome.
