# Supplementary material for: Comparative Analysis of Chloroplast Genomes of “Tiantai Wu-Yao” (Lindera aggregata) and Taxa of the Same Genus and Different Genera
Source: Genes (Basel). 2024 Feb 20;15(3):263. doi: 10.3390/genes15030263 (PMC10970223; doi:10.3390/genes15030263)
Supplement: Supplementary file 1 [file genes-15-00263-s001.zip › Figure S1-S3.pdf]

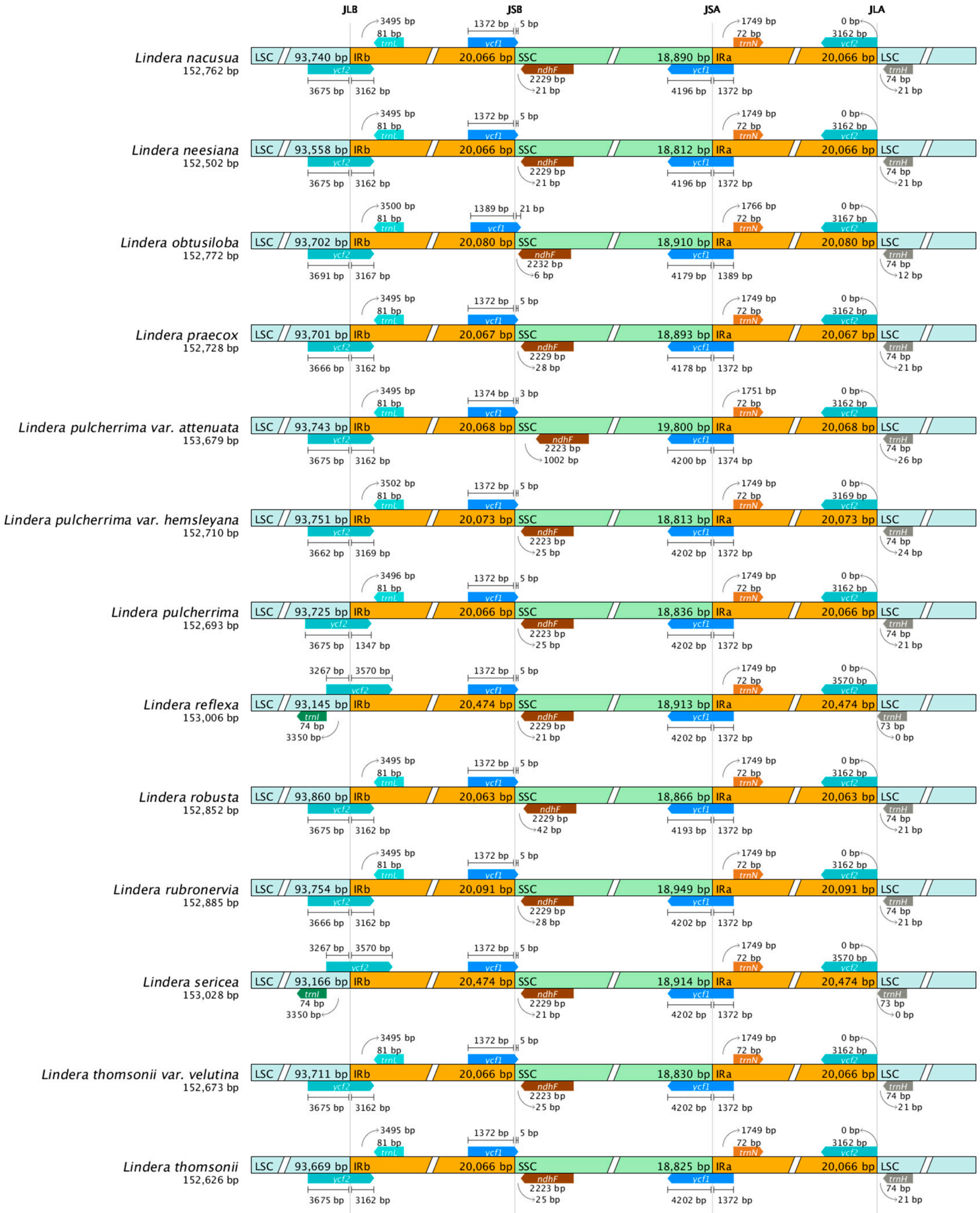

Figure S1 Comparison of junction boundaries in four regions (LSC/SSC/IRa/IRb) of 13 chloroplast genomes. The number on the color gene indicates the distance between the gene and the edge of the border. JLB represents LSC/IRb boundary, JSB represents SSC/IRb boundary, JSA represents SSC/IRa boundary, and JLA represents LSC/IRa boundary.

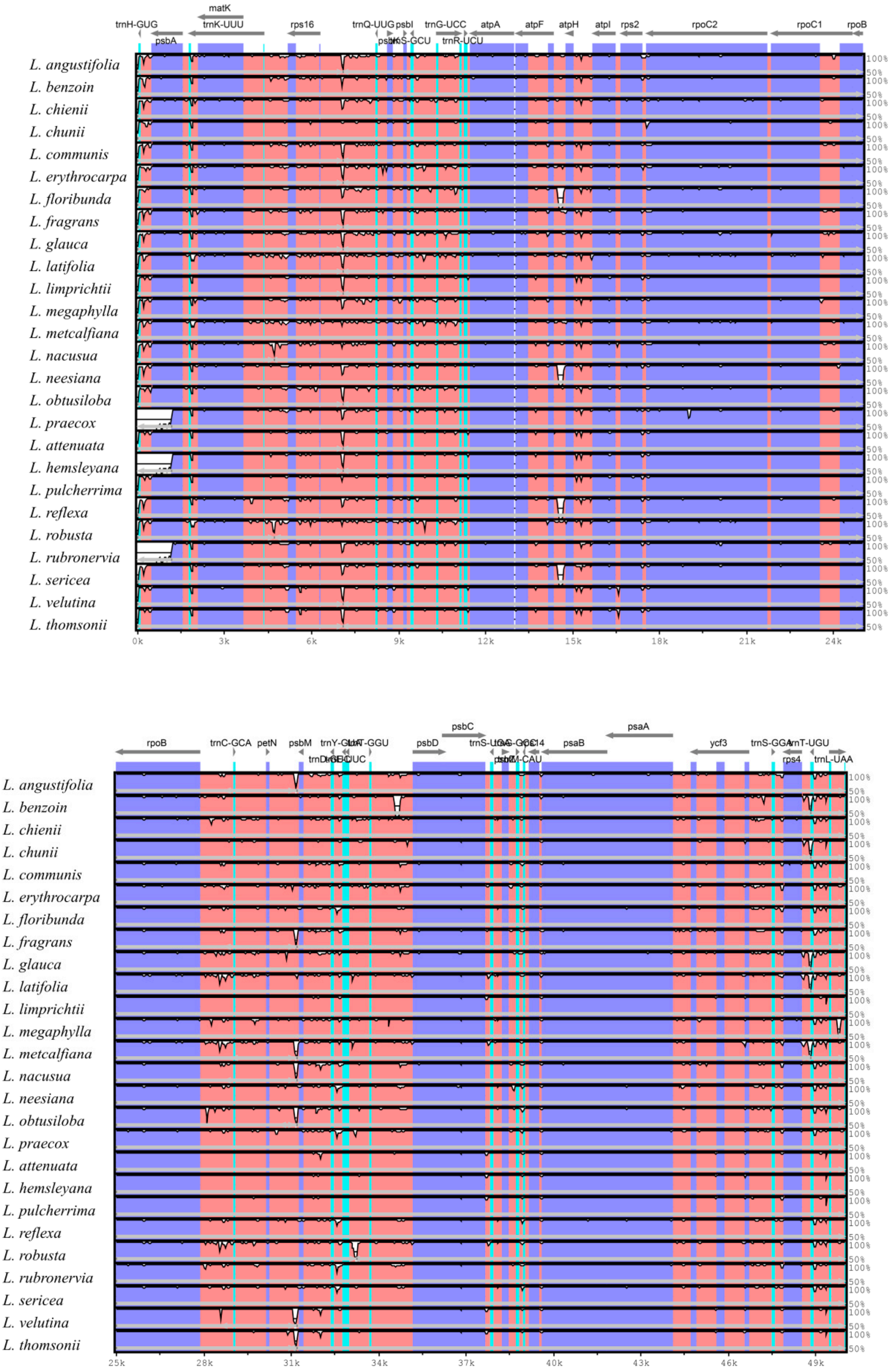

matK
trnH-GUG
psbA
trnK-UUU
rps16
trnQ-UUG
psbI
trnS-GCU
trnG-UCC
trnR-UCU
atpA
atpF
atpH
atpI
rps2
rpoC2
rpoC1
rpoB
L. angustifolia
L. benzoin
L. chienii
L. chunii
L. communis
L. erythrocarpa
L. floribunda
L. fragrans
L. glauca
L. latifolia
L. limprichtii
L. megaphylla
L. metcalfiana
L. nacusua
L. neesiana
L. obtusiloba
L. praecox
L. attenuata
L. hemsleyana
L. pulcherrima
L. reflexa
L. robusta
L. rubronervia
L. sericea
L. velutina
L. thomsonii
100%
50%
0k
3k
6k
9k
12k
15k
18k
21k
24k
rpoB
trnC-GCA
petN
psbM
trnY-GUA
trnT-GGU
psbD
psbC
psaB
psaA
ycf3
trnS-GGA
trnT-UGU
rps4
trnL-UAA
rps14
trnM-CAU
L. angustifolia
L. benzoin
L. chienii
L. chunii
L. communis
L. erythrocarpa
L. floribunda
L. fragrans
L. glauca
L. latifolia
L. limprichtii
L. megaphylla
L. metcalfiana
L. nacusua
L. neesiana
L. obtusiloba
L. praecox
L. attenuata
L. hemsleyana
L. pulcherrima
L. reflexa
L. robusta
L. rubronervia
L. sericea
L. velutina
L. thomsonii
100%
50%
25k
28k
31k
34k
37k
40k
43k
46k
49k

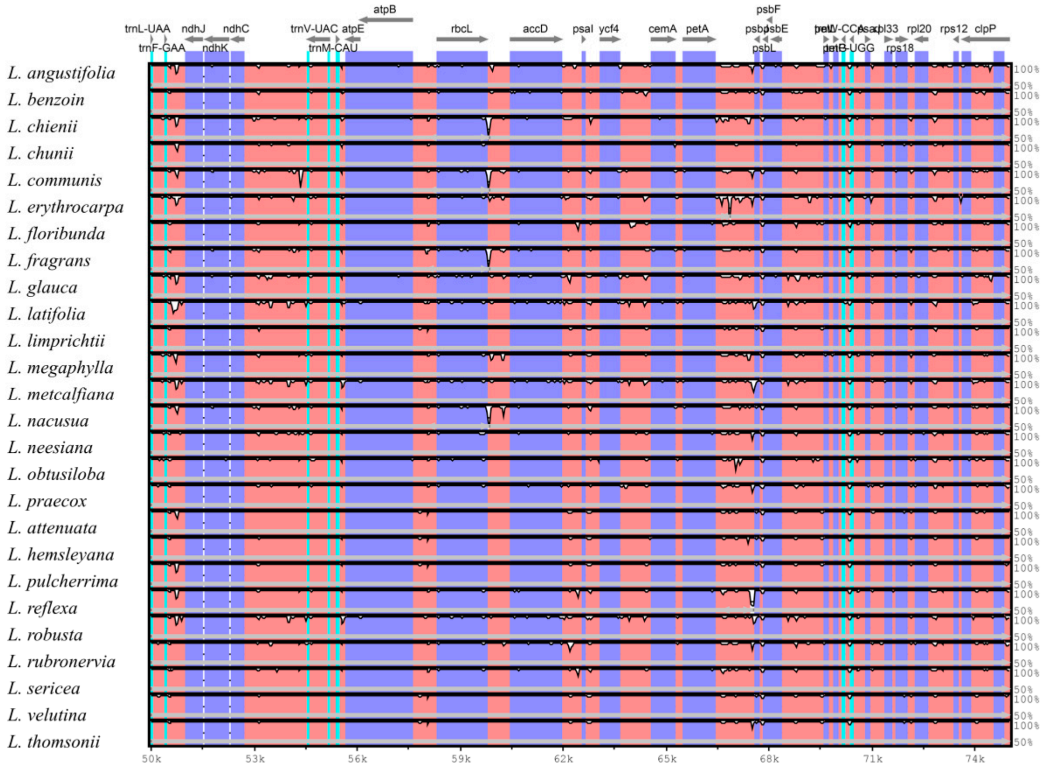

atpB
psbF
trnL-UAA
ndhJ
ndhC
trnV-UAC
atpE
rbcL
accD
psaI
ycf4
cemA
petA
psbE
rpl33
rpl20
rps12
clpP
trnF-GAA
ndhK
trnM-CAU
psbL
rps18
L. angustifolia
L. benzoin
L. chienii
L. chunii
L. communis
L. erythrocarpa
L. floribunda
L. fragrans
L. glauca
L. latifolia
L. limprichtii
L. megaphylla
L. metcalfiana
L. nacusua
L. neesiana
L. obtusiloba
L. praecox
L. attenuata
L. hemsleyana
L. pulcherrima
L. reflexa
L. robusta
L. rubronervia
L. sericea
L. velutina
L. thomsonii
100%
50%
50k
53k
56k
59k
62k
65k
68k
71k
74k

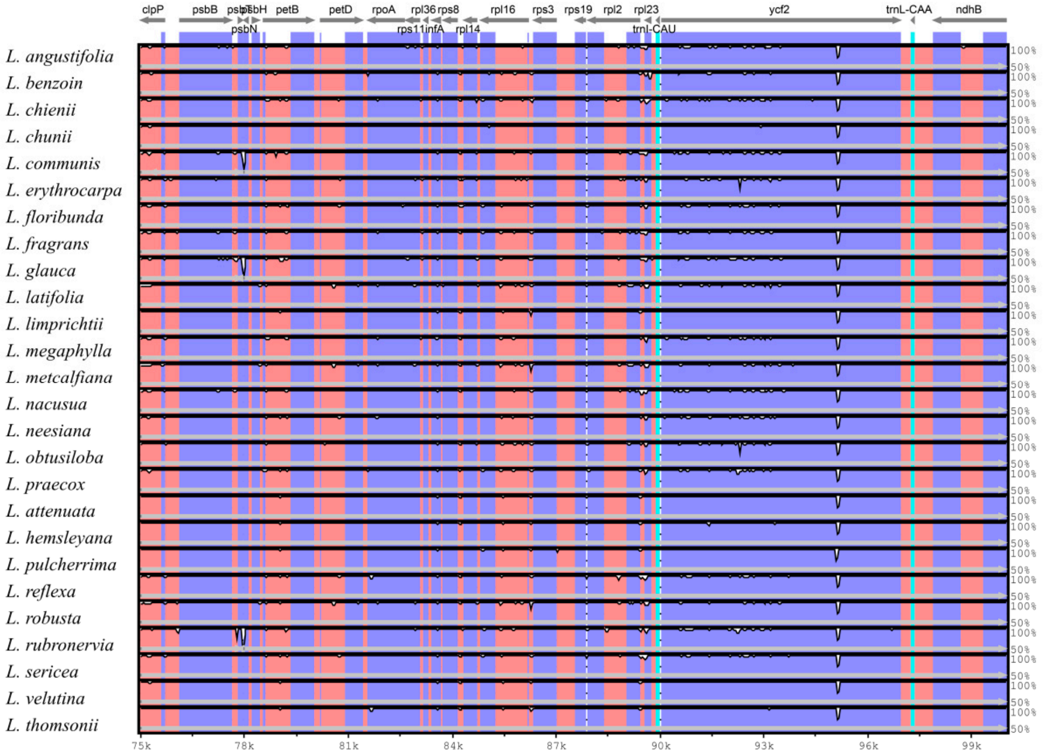

clpP
psbB
psbH
petB
petD
rpoA
rpl36
rps8
rpl16
rps3
rps19
rpl2
rpl23
ycf2
trnL-CAA
ndhB
psbN
rps11
infA
rpl14
trnI-CAU
L. angustifolia
L. benzoin
L. chienii
L. chunii
L. communis
L. erythrocarpa
L. floribunda
L. fragrans
L. glauca
L. latifolia
L. limprichtii
L. megaphylla
L. metcalfiana
L. nacusua
L. neesiana
L. obtusiloba
L. praecox
L. attenuata
L. hemsleyana
L. pulcherrima
L. reflexa
L. robusta
L. rubronervia
L. sericea
L. velutina
L. thomsonii
100%
50%
75k
78k
81k
84k
87k
90k
93k
96k
99k

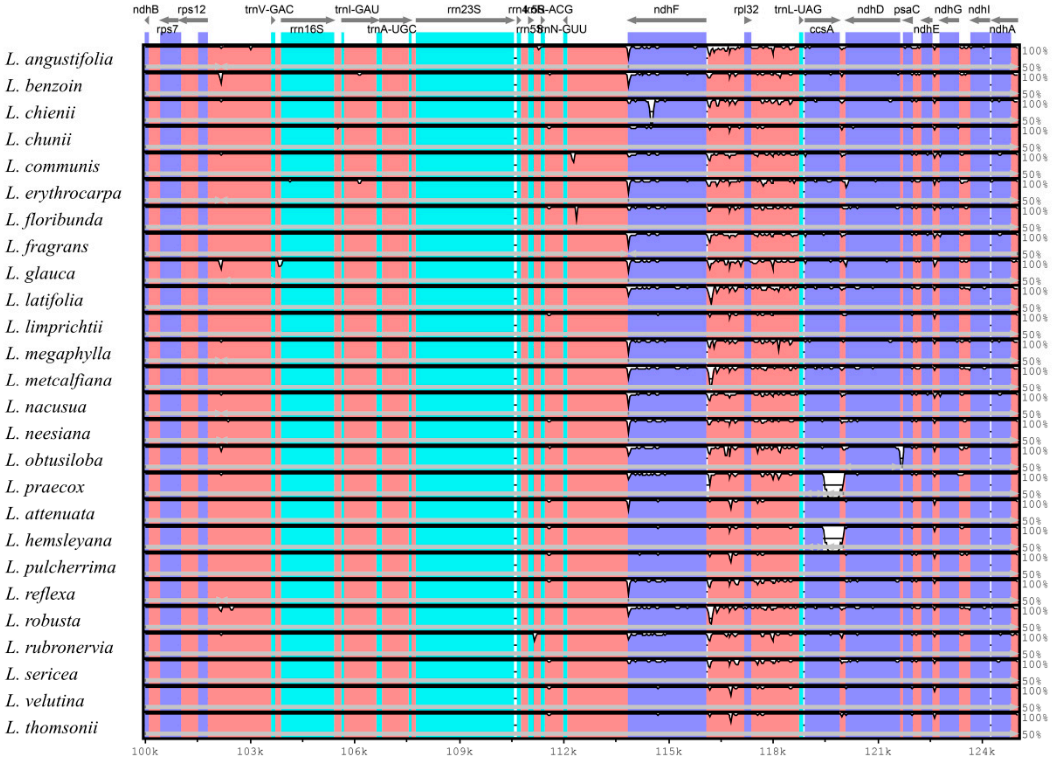
ndhB
rps12
rps7
trnV-GAC
rrn16S
trnI-GAU
trnA-UGC
rrn23S
trnN-GUU
ndhF
rpl32
trnL-UAG
ccsA
ndhD
psaC
ndhE
ndhG
ndhI
ndhA
L. angustifolia
L. benzoin
L. chienii
L. chunii
L. communis
L. erythrocarpa
L. floribunda
L. fragrans
L. glauca
L. latifolia
L. limprichtii
L. megaphylla
L. metcalfiana
L. nacusua
L. neesiana
L. obtusiloba
L. praecox
L. attenuata
L. hemsleyana
L. pulcherrima
L. reflexa
L. robusta
L. rubronervia
L. sericea
L. velutina
L. thomsonii
100%
50%
100k
103k
106k
109k
112k
115k
118k
121k
124k

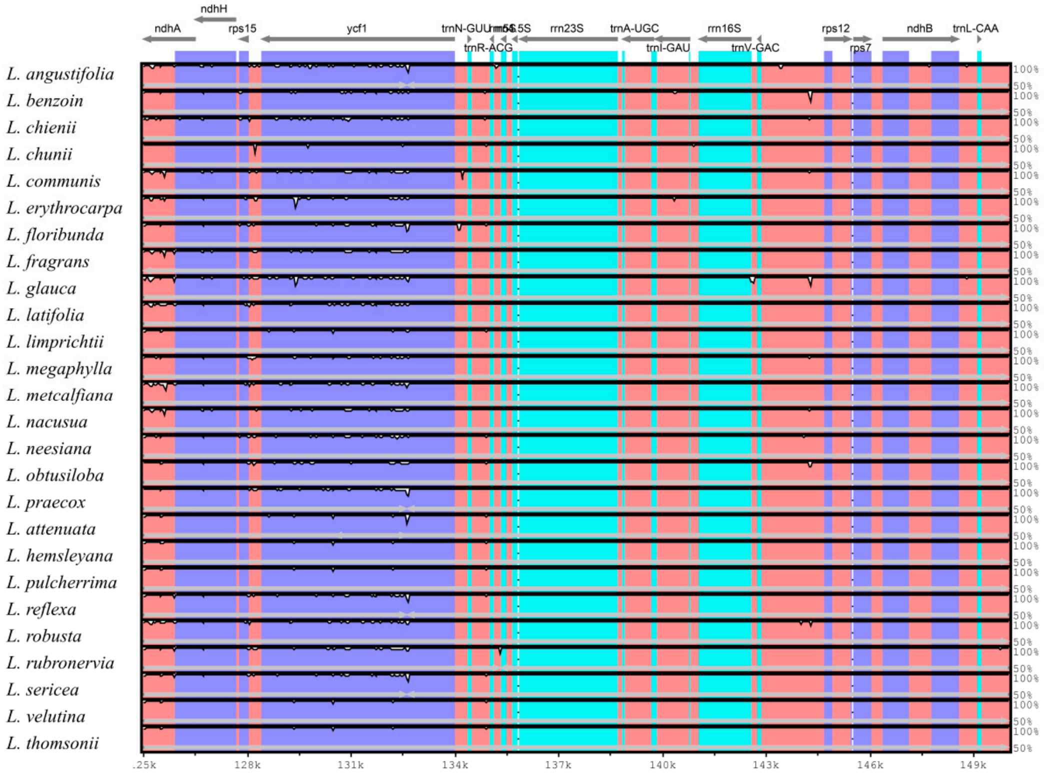
ndhH
ndhA
rps15
ycf1
trnN-GUU
trnR-ACG
rrn23S
trnA-UGC
trnI-GAU
rrn16S
trnV-GAC
rps12
rps7
ndhB
trnL-CAA
L. angustifolia
L. benzoin
L. chienii
L. chunii
L. communis
L. erythrocarpa
L. floribunda
L. fragrans
L. glauca
L. latifolia
L. limprichtii
L. megaphylla
L. metcalfiana
L. nacusua
L. neesiana
L. obtusiloba
L. praecox
L. attenuata
L. hemsleyana
L. pulcherrima
L. reflexa
L. robusta
L. rubronervia
L. sericea
L. velutina
L. thomsonii
100%
50%
128k
131k
134k
137k
140k
143k
146k
149k

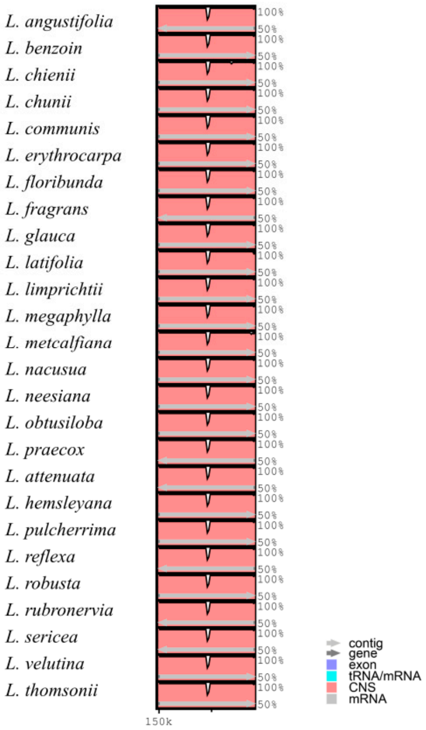

Figure S2 The sequence difference map compares 26 chloroplast genomes of *Lindera*, using the chloroplast genome of *L. aggregata* as the reference sequence via mVISTA software. The gray arrows and thick black lines above indicate the direction of the gene. Different areas were given different colors. The pink region was a conservative non-coding region (CNS), purple was the gene exon region, and the blue region was tRNA or rRNA.

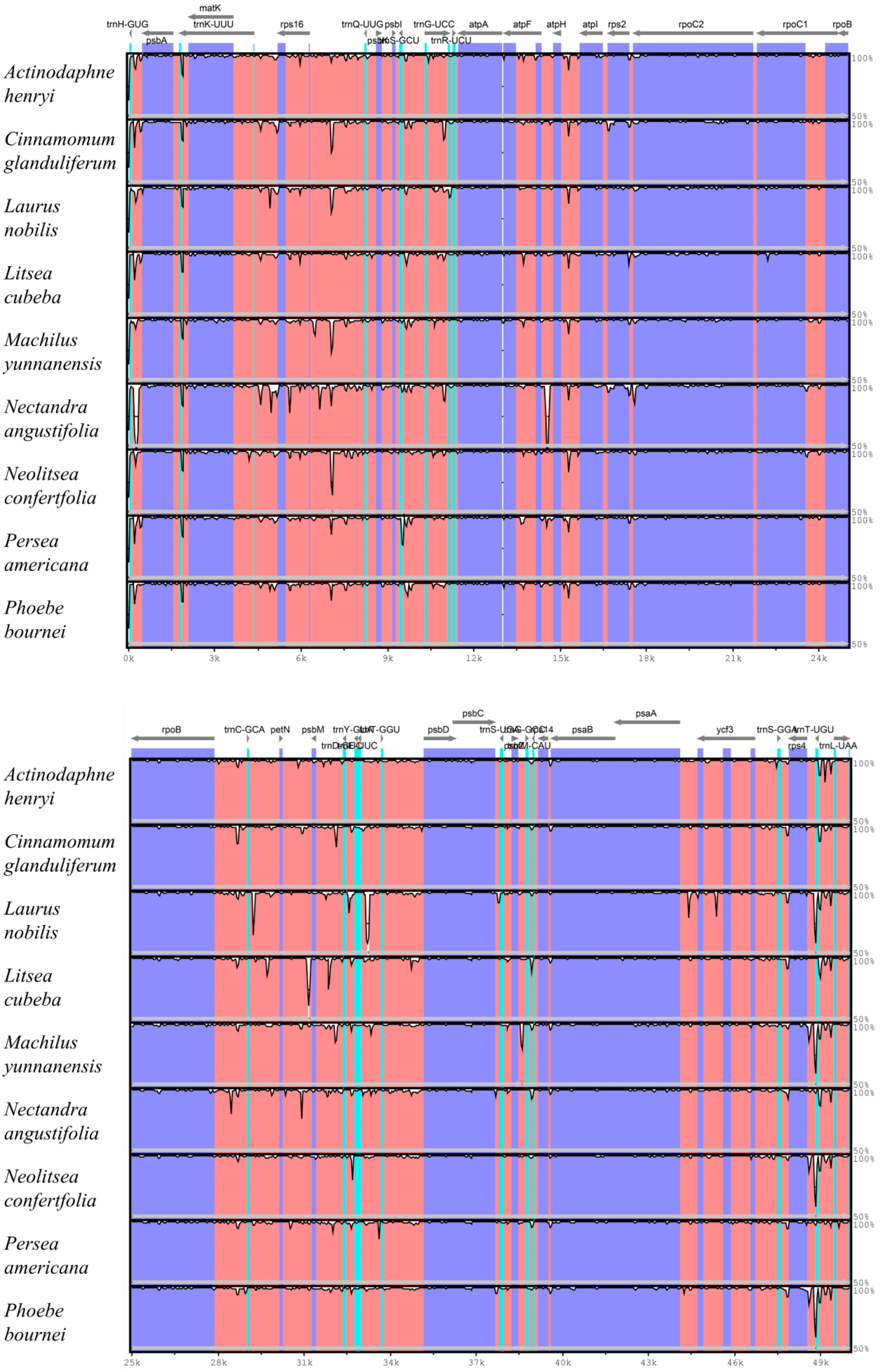

Actinodaphne henryi
Cinnamomum glanduliferum
Laurus nobilis
Litsea cubeba
Machilus yunnanensis
Nectandra angustifolia
Neolitsea confertfolia
Persea americana
Phoebe bournei
matK
trnH-GUG
trnK-UUU
psbA
rps16
trnQ-UUG
psbI
trnG-UCC
trnR-UCU
atpA
atpF
atpH
atpI
rps2
rpoC2
rpoC1
rpoB
100%
50%
0k
3k
6k
9k
12k
15k
18k
21k
24k
psbC
psaA
rpoB
trnC-GCA
petN
psbM
trnT-GGU
psbD
psaB
ycf3
trnT-UGU
rps4
trnL-UAA
25k
28k
31k
34k
37k
40k
43k
46k
49k

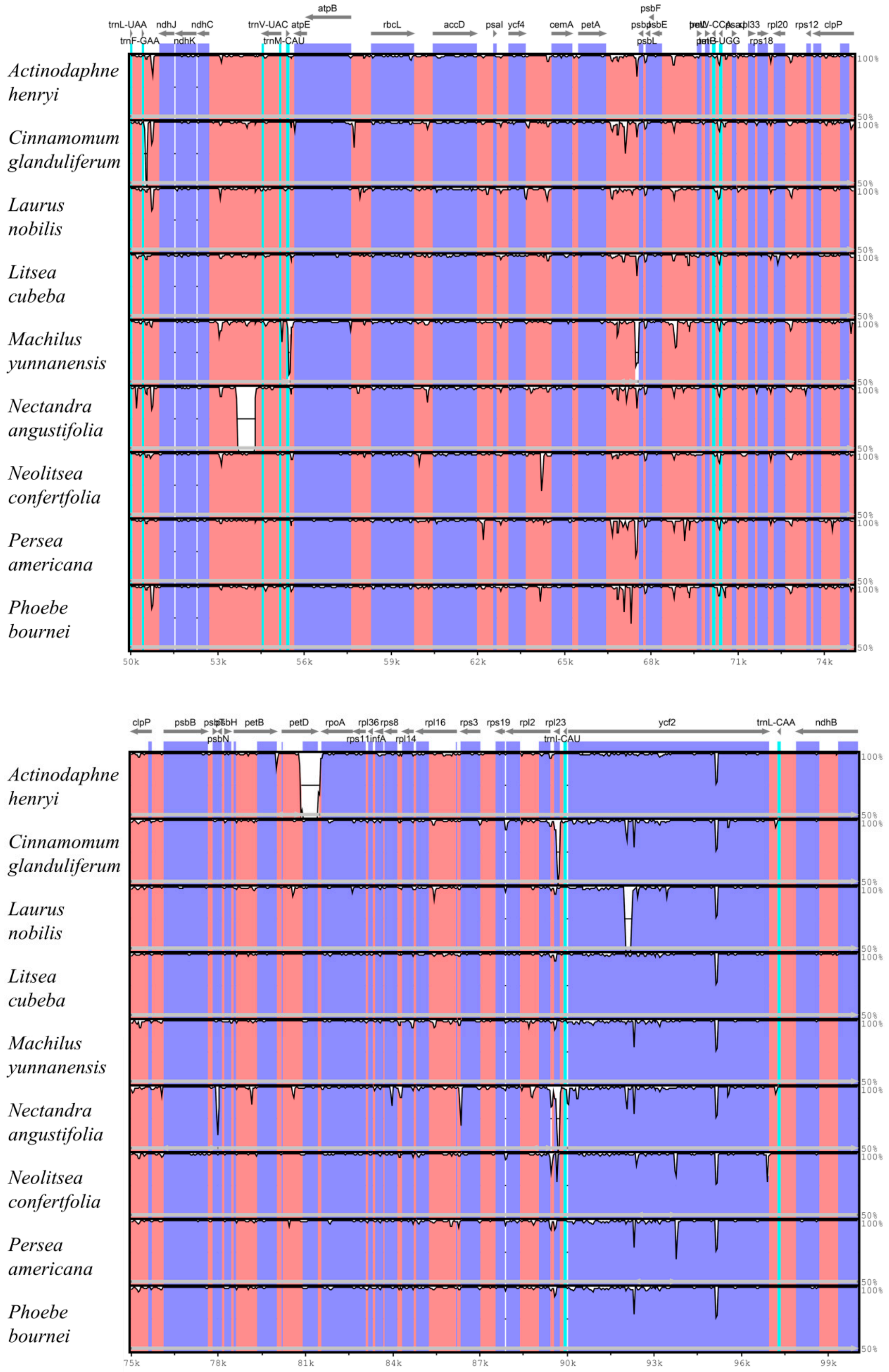

trnL-UAA
trnF-GAA
ndhJ
ndhK
ndhC
trnV-UAC
trnM-CAU
atpE
atpB
rbcL
accD
psaI
ycf4
cemA
petA
psbF
psbE
psbL
rpl33
rps18
rpl20
rps12
clpP
Actinodaphne henryi
Cinnamomum glanduliferum
Laurus nobilis
Litsea cubeba
Machilus yunnanensis
Nectandra angustifolia
Neolitsea confertfolia
Persea americana
Phoebe bournei
100%
50%
50k
53k
56k
59k
62k
65k
68k
71k
74k
clpP
psbB
psbH
psbN
petB
petD
rpoA
rps11
rpl36
infA
rps8
rpl14
rpl16
rps3
rps19
rpl2
rpl23
trnI-CAU
ycf2
trnL-CAA
ndhB
Actinodaphne henryi
Cinnamomum glanduliferum
Laurus nobilis
Litsea cubeba
Machilus yunnanensis
Nectandra angustifolia
Neolitsea confertfolia
Persea americana
Phoebe bournei
100%
50%
75k
78k
81k
84k
87k
90k
93k
96k
99k

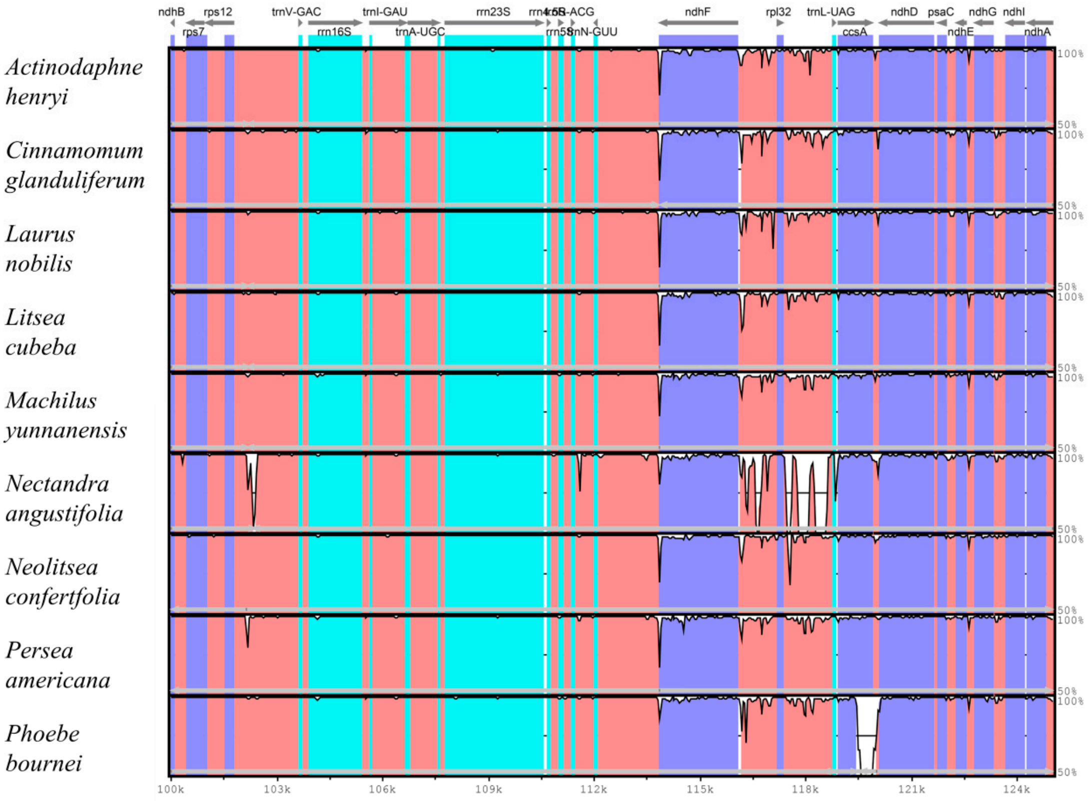

ndhB
rps7
rps12
trnV-GAC
rrn16S
trnI-GAU
trnA-UGC
rrn23S
ndhF
rpl32
trnL-UAG
ccsA
ndhD
psaC
ndhE
ndhG
ndhI
ndhA
Actinodaphne henryi
Cinnamomum glanduliferum
Laurus nobilis
Litsea cubeba
Machilus yunnanensis
Nectandra angustifolia
Neolitsea confertfolia
Persea americana
Phoebe bournei
100%
50%
100k
103k
106k
109k
112k
115k
118k
121k
124k

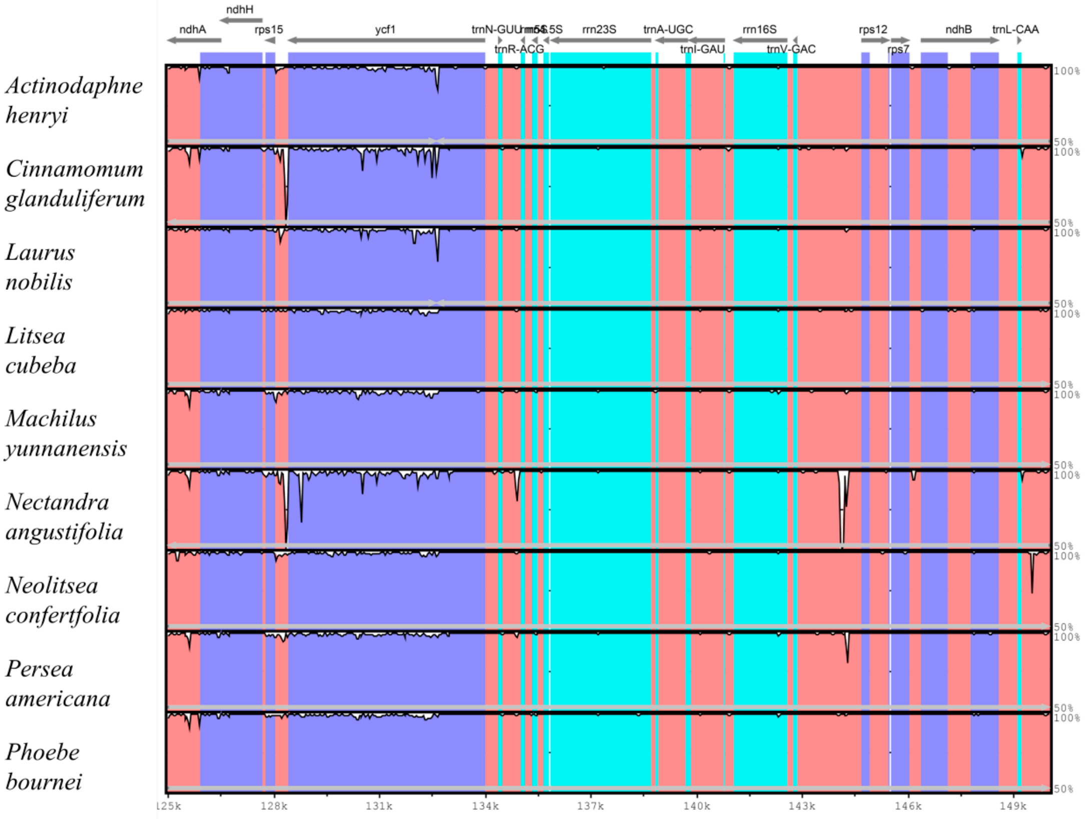

ndhH
ndhA
rps15
ycf1
trnR-ACG
rrn23S
trnA-UGC
trnI-GAU
rrn16S
trnV-GAC
rps12
rps7
ndhB
trnL-CAA
Actinodaphne henryi
Cinnamomum glanduliferum
Laurus nobilis
Litsea cubeba
Machilus yunnanensis
Nectandra angustifolia
Neolitsea confertfolia
Persea americana
Phoebe bournei
100%
50%
128k
131k
134k
137k
140k
143k
146k
149k

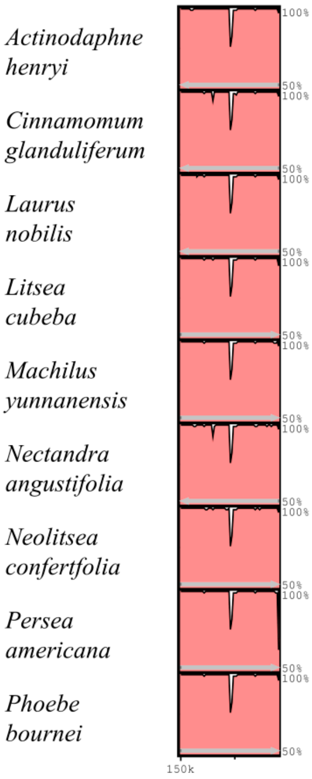

Figure S3 The sequence difference map compares 9 chloroplast genomes of Lauraceae, using the chloroplast genome of *L. aggregata* as the reference sequence via mVISTA software. The gray arrows and thick black lines above indicate the direction of the gene. Different areas were given different colors. The pink region was a conservative non-coding region (CNS), purple was the gene exon region, and the blue region was tRNA or rRNA.
